# Repeat traumatic brain injury exacerbates acute thalamic hyperconnectivity in humans

**DOI:** 10.1093/braincomms/fcae223

**Published:** 2024-06-28

**Authors:** Rebecca E Woodrow, David K Menon, Emmanuel A Stamatakis, CENTER-TBI MRI Sub-study Participants and Investigators, Krisztina Amrein, Nada Andelic, Lasse Andreassen, Audny Anke, Philippe Azouvi, BoMichael Bellander, Habib Benali, Andras Buki, Alessio Caccioppola, Emiliana Calappi, Marco Carbonara, Giuseppe Citerio, Hans Clusmann, Mark Coburn, Jonathan Coles, Marta Correia, Endre Czeiter, Véronique De Keyser, Vincent Degos, Bart Depreitere, Live Eikenes, Erzsébet Ezer, Kelly Foks, Shirin Frisvold, Damien Galanaud, Alexandre Ghuysen, Ben Glocker, Asta Haberg, Iain Haitsma, Eirik Helseth, Peter J Hutchinson, Evgenios Kornaropoulos, Noémi Kovács, Ana Kowark, Steven Laureys, Didier Ledoux, Hester Lingsma, Andrew I R Maas, Geoffrey Manley, David K Menon, Tomas Menovsky, Benoit Misset, Visakh Muraleedharan, Ingeborg Nakken, Virginia Newcombe, Wibeke Nordhøy, József Nyirádi, Fabrizio Ortolano, Paul M Parizel, Vincent Perlbarg, Paolo Persona, Wilco Peul, Jussi P Posti, Louis Puybasset, Sophie Richter, Cecilie Roe, Olav Roise, Rolf Rossaint, Sandra Ross, Daniel Rueckert, Ranjit D Singh, Toril Skandsen, Abayomi Sorinola, Emmanuel Stamatakis, Ewout W Steyerberg, Nino Stocchetti, Riikka Takala, Viktória Tamás, Olli Tenovuo, Aurore Thibaut, Zoltán Vámos, Gregory Van der Steen, Inge A van Erp, Wim Van Hecke, Thijs Vande Vyvere, Jan Verheyden, Anne Vik, Victor Volovici, Lars T Westlye, Daniel Whitehouse, Guy Williams, Stefan Winzeck, Peter Ylén, Tommaso Zoerle

**Affiliations:** University Division of Anaesthesia, University of Cambridge, Cambridge CB2 0SP, UK; Department of Clinical Neurosciences, University of Cambridge, Cambridge CB2 0QQ, UK; University Division of Anaesthesia, University of Cambridge, Cambridge CB2 0SP, UK; Wolfson Brain Imaging Centre, University of Cambridge, Cambridge CB2 0QQ, UK; University Division of Anaesthesia, University of Cambridge, Cambridge CB2 0SP, UK

**Keywords:** mild traumatic brain injury, concussion, fMRI, neuroimaging, thalamus

## Abstract

Repeated mild traumatic brain injury is of growing interest regarding public and sporting safety and is thought to have greater adverse or cumulative neurological effects when compared with single injury. While epidemiological links between repeated traumatic brain injury and outcome have been investigated in humans, exploration of its mechanistic substrates has been largely undertaken in animal models. We compared acute neurological effects of repeat mild traumatic brain injury (*n* = 21) to that of single injury (*n* = 21) and healthy controls (*n* = 76) using resting-state functional MRI and quantified thalamic functional connectivity, given previous identification of its prognostic potential in human mild traumatic brain injury and rodent repeat mild traumatic brain injury. Acute thalamocortical functional connectivity showed a rank-based trend of increasing connectivity with number of injuries, at local and global scales of investigation. Thus, history of as few as two previous injuries can induce a vulnerable neural environment of exacerbated hyperconnectivity, in otherwise healthy individuals from non-specialist populations. These results further establish thalamocortical functional connectivity as a scalable marker of acute injury and long-term neural dysfunction following mild traumatic brain injury.

## Teaser

Woodrow *et al*. compared acute neurological effects of repeat mild traumatic brain injury to that of single and no injury using resting-state functional MRI. Acute thalamocortical functional connectivity showed a rank-based trend of increasing connectivity with number of injuries, indicating a vulnerable neural environment in non-specialist populations.

## Graphical Abstract

**Graphical Abstract:**
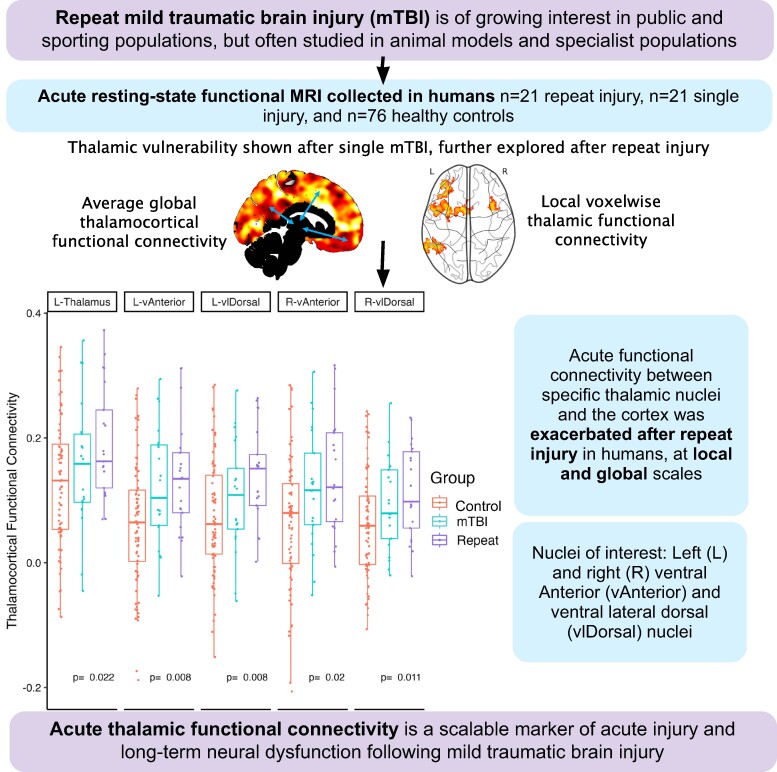

## Introduction

Repeat mild traumatic brain injury (mTBI) can manifest greater behavioural and neurological changes than a single injury.^1-3^ Comparatively, history of multiple concussions can increase the number and persistence of post-concussive symptoms (PCSs) after subsequent injury,^4,5^ including increased susceptibility to lifelong neurodegenerative disease.^6^ It is thus of great public interest to understand neurological and safety consequences of repeat mTBI, which are increasingly recognized as a common occurrence in contact sports.

The thalamus has been highlighted as a region of interest given its vulnerable location to primary injury.^7^ For example, collegiate footballers showed acute white matter damage of thalamocortical pathways^8^ and chronic loss of thalamic anticorrelation that worsened with time since play in retired National Football League players.^9^ Additionally, mouse models have shown greatest early levels of neuroinflammation in the thalamus out of any brain region^10^ and increased calcifications 4-week post-injury compared to those suffering a single mTBI.^11^ Thus, thalamic injury may be particularly amplified after repeat mTBI at both acute and chronic timepoints.

We previously identified acute thalamocortical hyperconnectivity in single mTBI, associated with high rates of adverse post-concussive outcome.^12^ Thalamic hyperconnectivity has subsequently been replicated as a prognostic marker, linked to loss of inhibitory control and exacerbated in a repeat mTBI rat model.^13^ In this study, rats exposed to two mTBIs experienced greater changes in microstructural white matter damage surrounding the thalami, greater markers of thalamic neuroinflammation and prolonged periods of thalamic hyperconnectivity compared to rats exposed to one mTBI. These controlled animal models^13,14^ are integral to our understanding of repeat versus single mTBI, but its effects are yet to be fully explored in humans using neuroimaging.

We capitalized on the unique multicentre data set CENTER-TBI^15^ to establish a single versus multiple injury analysis cohort in humans—not previously been investigated with functional MRI (fMRI). To probe acute effects of repeat injury, we considered control, single mTBI and repeat injury groups simultaneously using resting-state fMRI, hypothesized to observe increasing acute thalamocortical connectivity across the three groups. Uniquely, this investigated members of the public rather than specific sporting communities, undergoing a mere handful of previous injuries, to better understand cumulative effects of repetitive TBI and implications for public policy.

## Materials and methods

Individuals were identified from CENTER-TBI (vCORE 3.0), aged 18–70 years with no medical history of neuropsychiatric disease, as defined previously.^12^ Patients sustained a mTBI (Glasgow Coma Scale 13–15), required a head CT according to local criteria on initial presentation, showed no CT abnormalities, were not treated in the intensive care unit and had both 3T T_1_-weighted MRI (1 mm3) and resting-state fMRI (3 mm3) in the acute phase post-injury. Acquisition protocols for these imaging data are described in the central CENTER-TBI resources at https://www.center-tbi.eu/project/mri-study-protocols. CENTER-TBI obtained ethical approval (https://www.center-tbi.eu/project/ethical-approval), and informed consent was given by each participant/legal representative.

Additional criterion for the repeat group was history of two or more previous TBIs, defined using medical history of ‘total pre-injury concussions/TBI’ or self-reported ‘pre-injury sports-related concussions/TBI’ obtained 6 months post injury, using self-report as the gold standard. This identified *n* = 21 participants with history of TBI, *n* = 11 with 2 previously reported injuries, up to 5 previous injuries in one participant. As none demonstrated damage on CT, we infer these prior injuries were likely consistent with concussion/mTBI. We identified *n* = 76 healthy controls, and age- and sex-matched a single mTBI group, for *n* = 21 participants.

The repeat TBI group and controls were compared in age (Fisher’s exact), and sex (*χ*^2^). Tests were chosen to account for the categorical nature of age recruitment in CENTER-TBI protocols, which aim to combat possible differences in admission rates during study recruitment. However, age is hereafter treated as a continuous covariate in all statistical analyses. TBI groups were also compared using Fisher’s exact test in baseline Glasgow Coma Scale, and 6-month outcomes of ‘complete’ [Glasgow Outcome Scale Extended (GOSE)-8] versus ‘incomplete’ (GOSE ≤7) recovery, and post-concussion symptom (PCS) positive or negative according to ICD-10 criteria of presenting three or more specified symptoms on the Rivermead Post-Concussion Questionnaire.

Preprocessing of neuroimaging data used fmriprep,^16^ with functional data denoised by signal regression, motion censoring and spatial smoothing (6 mm Gaussian kernel) replicating our previous methods,^12^ detailed in Supplementary Methods 1. All data were statistically harmonized across site/scanner using ComBat^17^ prior to group comparisons, preserving biological covariates of age, sex and clinical group.

### Statistical analysis

Groups were considered simultaneously in two analyses—global thalamocortical connectivity and voxelwise thalamic connectivity—for the left and right thalamus and seven thalamic nuclei per hemisphere.^18^ Mean thalamocortical functional connectivity was calculated for each region of interest (ROI) (*n* = 16) for each subject, using beta-maps of ROI-to-voxel functional connectivity using the CONN toolbox v.20.b, and a mean connectivity was calculated across each individual’s cortical grey matter mask. These metrics were analysed using a one-tailed Jonckheere–Terpstra test for non-parametric rank-based trends, with *N* = 1000 permutations, hypothesized to observe increasing thalamocortical connectivity across the three groups (control, single mTBI and repeat). All tests were adjusted for effects of age and sex and FDR-corrected at *P* < 0.05. Significant variables were further investigated between the single mTBI and repeat groups using a one-tailed exact Jonckheere–Terpstra test.

ROI-to-voxel beta maps for *n* = 16 thalamic ROIs were subsequently investigated for voxelwise connectivity differences between groups, using SPM12 in a linear regression. These tests were conducted with covariates of age and sex, at voxel-level *P* < 0.001 (uncorrected) and cluster-level family-wise error-corrected *P* < 0.05. Significant thalamic seeds were further investigated using a two-sample *t*-test between single mTBI and repeat groups according to the same statistical criteria.

## Results

Our cohort included *n* = 21 repeat mTBI, *n* = 21 age- and sex-matched single mTBI and *n* = 76 healthy control participants (consort diagram for inclusion presented in Supplementary Fig. 1). TBI participants had a mean age of 34.3 years (SD 13.9) at time of injury, and controls had a mean age of 42.2 years (SD 11.7). Repeat TBI and control groups did not differ in age [*X*^2^(1) = 4.5, *P* = 0.11] or sex [*X*^2^(1) = 0.01, *P* = 0.91]. Single mTBI and repeat groups underwent neuroimaging at a mean of 12.5 ± 9.9 days post-injury and showed no statistically significant differences in severity of the index injury that triggered recruitment to CENTER-TBI (Fisher’s exact *P* = 1.0) or 6-month outcome in GOSE or PCS presentation (Fisher’s exact *P* = 1.0; *P* = 0.71), respectively. Within the repeat group, *n* = 16 reported a history of sports-related concussion/TBI: skiing (*n* = 4), ice hockey/skating (*n* = 3), horse riding (*n* = 2), gymnastics (*n* = 2), handball (*n* = 2) and football (*n* = 2). Further information for all groups is presented in Table 1.

**Table 1:** Demographic and clinical characteristics

	Control (*n* = 76)	Single mTBI (*n* = 21)	Repeat (*n* = 21)	
	*n* (%)	*n* (%)	*n* (%)	
				Repeat versus control
Age				
18–35	26 (34.2)	12 (57.1)	12 (57.1)	*X* ^2^(1) = 4.5, *P* = 0.11
36–55	36 (47.4)	8 (38.1)	8 (38.1)	
55–70	14 (18.4)	1 (4.8)	1 (4.8)	
Sex				
Male	46 (60.5)	13 (61.9)	13 (61.9)	*X* ^2^(1) = 0.01, *P* = 0.91
Female	30 (39.5)	8 (38.1)	8 (38.1)	
				Single mTBI versus repeat
Glasgow Coma Score				
15		18 (85.7)	18 (85.7)	Fisher’s exact *P* = 1.0
14		2 (9.5)	3 (14.3)	
13		1 (4.8)	0 (0)	
Injury cause				
Road traffic incident		11 (52.4)	8 (38.1)	Fisher’s exact *P* = 0.86
Incidental fall		6 (28.6)	7 (33.3)	
Other non-intentional injury		2 (9.5)	2 (9.5)	
Violence/assault		2 (9.5)	3 (14.3)	
Act of mass violence		0 (0)	0 (0)	
Other		0 (0)	1 (4.8)	
Strata				
Emergency room		11 (52.4)	11 (52.4)	Fisher’s exact *P* = 1.0
Admission		10 (47.6)	10 (47.6)	
6-month GOSE		*n* = 20	*n* = 20	Fisher’s exact *P* = 1.0
Complete		12 (60.0)	13 (65.0)	
Incomplete		8 (40.0)	7 (45.0)	
6-month PCS		*n* = 18	*n* = 20	Fisher’s exact *P* = 0.70
PCS+		5 (27.8)	4 (20.0)	
PCS−		13 (72.2)	16 (80.0)	

Data are presented for healthy controls, repeat and the age + sex-matched single mTBI group. Numbers of participants included per group are indicated by *n*.

**Figure 1:**
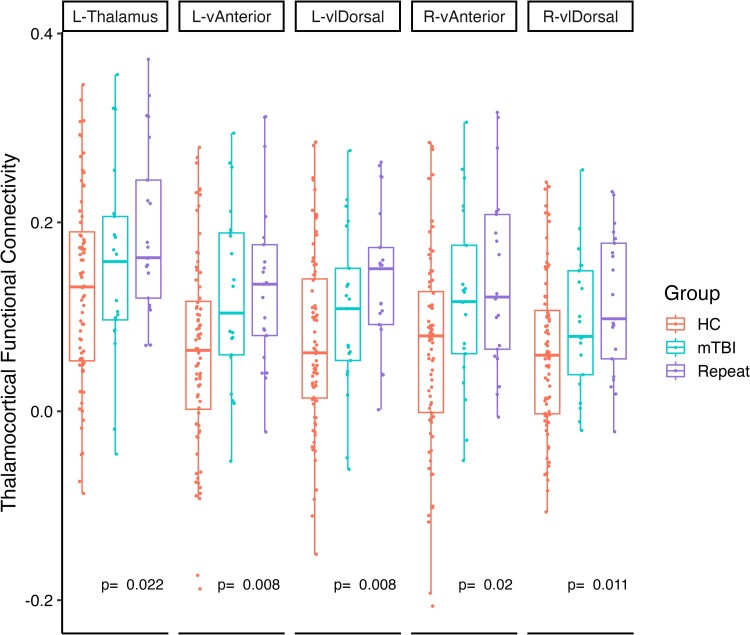
**Globally increasing thalamocortical functional connectivity.** Thalamic seeds showing significantly greater global connectivity with an increasing number of concussive events, assessed using one-tailed Jonckheere–Terpstra test for non-parametric rank-based trends with *N* = 1000 permutations. Nuclei shown are left thalamus (L-Thalamus), left and right ventral anterior (L-vAnterior, R-vAnterior) and left and right ventral lateral dorsal (L-lDorsal, R-vlDorsal). Each dot indicates an individual subject within that group, of healthy control (HC), single injury (mTBI) and repeat injury (Repeat). *P*-values are FDR-corrected for *n* = 16 comparisons.

Mean thalamocortical functional connectivity showed a significant rank-based increasing trend with number of TBIs across the three groups (control, single mTBI and repeat; Fig. 1) in the same thalamic nuclei we previously proposed to be vulnerable in mTBI^12^: the bilateral ventral anterior nuclei (vAnterior; left *T*_JT_ = 2399, *P* = 0.008; right *T*_JT_ = 2358, *P* = 0.011) and bilateral ventral lateral dorsal nuclei (vlDorsal; left *T*_JT_ = 2375, *P* = 0.008; right *T*_JT_ = 2291, *P* = 0.020). There was additionally a significant increase in connectivity to the cortex from the whole left thalamus (*T*_JT_ = 2244, *P* = 0.022). However, we did not observe significant differences in connectivity to the cortex for any of the specific ROIs between the single and repeat groups [left thalamus (*T*_JT_ = 265, *P* = 0.30); L-vlDorsal (*T*_JT_ = 267, *P* = 0.30); L-vAnterior (*T*_JT_ = 236, *P* = 0.35); R-vlDorsal (*T*_JT_ = 258, *P* = 0.30); R-vAnterior (*T*_JT_ = 238, *P* = 0.35)]. Results for all non-significant nuclei are presented in Supplementary Table 1.

**Figure 2:**
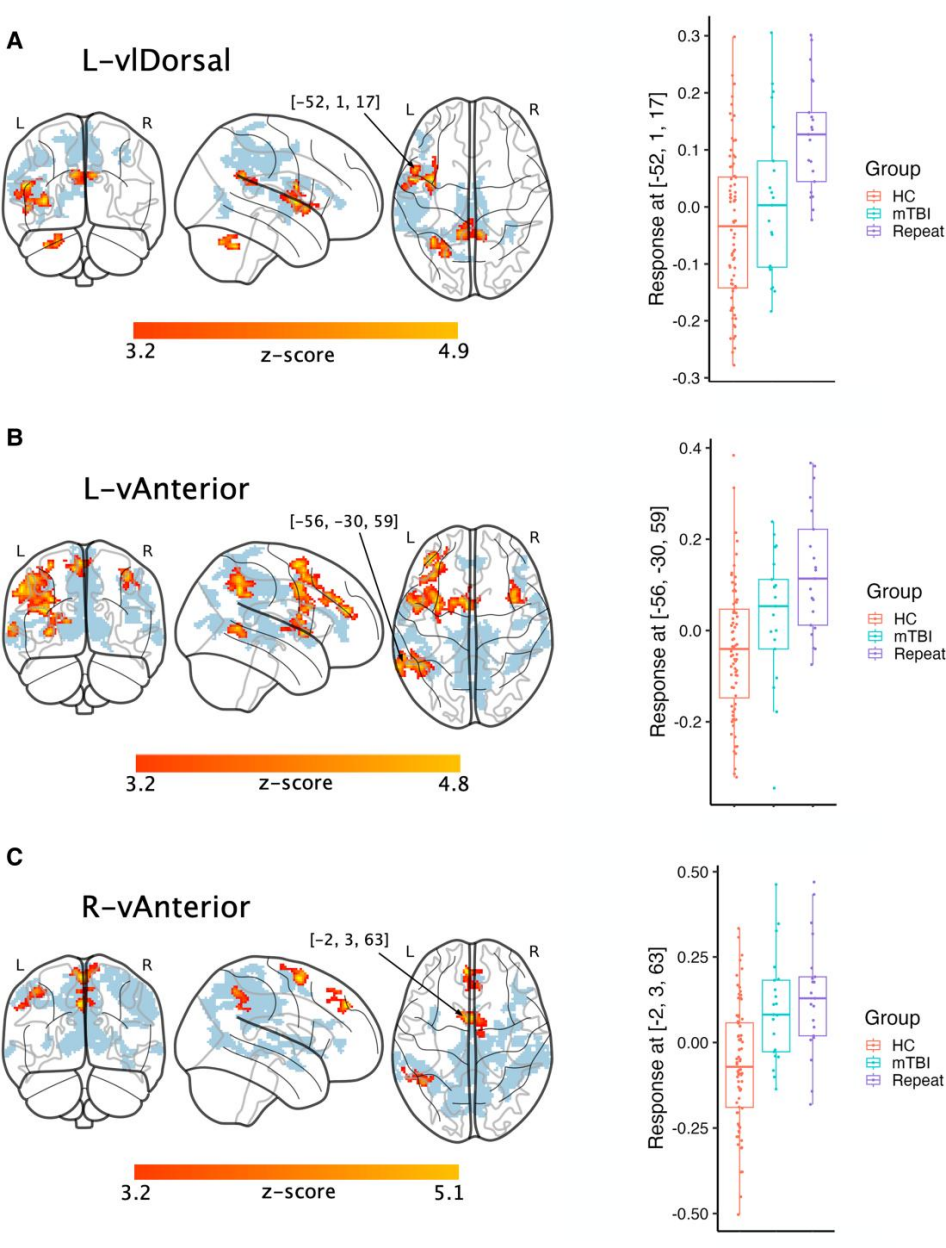
**Locally increasing thalamic functional connectivity.** Results from linear regression voxelwise analyses with covariates of sex and age, showing regions of linearly increasing thalamic connectivity from controls, to single mTBI, to repeat injury (*Z*-scores scale presented below slices). Significant regions are those surviving *P* < 0.001 (uncorrected) at the voxel level and family-wise error-corrected *P* < 0.05 at the cluster level. Regions shown shaded indicate those areas found to have significant differences between controls and single mTBI alone, as detailed in Woodrow *et al*.^12^  **A-C** show three independent nuclei, with their global maximum *Z*-score coordinate indicated with an arrow in the transverse slice. Individual responses of thalamic functional connectivity to this coordinate are plotted in the corresponding right-hand column, for visualization purposes only. Each dot indicates an individual subject within that group, for healthy control (HC), single injury (mTBI) and repeat injury (Repeat). Thalamic nuclei shown are the left ventral lateral dorsal (L-vlDorsal) and left and right ventral anterior (L-vAnterior, R-vAnterior).

Voxelwise linear regression analysis identified clusters of significant change in the same ‘vulnerable’ nuclei—bilateral vAnterior and right vlDorsal nuclei, as shown in Fig. 2. Functional connectivity between the thalamic ROI and its corresponding voxel coordinate displaying the strongest relationship to group membership was extracted for each participant and plotted by group to visually demonstrate stepwise linear increase in thalamic connectivity. No regions of significant difference were found when explicitly comparing single mTBI and repeat groups in any nuclei.

Interestingly, significant clusters identified as increasing acute thalamic connectivity with number of concussions partially overlapped previous regions found to be hyperconnected in single mTBI^12^ (Fig. 2), namely, 46.9% overlap between clusters with the left vlDorsal seed, 13.8% overlap with the left vAnterior seed and 33.9% overlap with the right vAnterior seed.

## Discussion

We explored effects of repeat TBI, in comparison to a single injury and no injury. This analysis was performed through the lens of the thalamus—previously shown to index both injury processes and outcome after a ‘mild’ TBI.^12^ We found that acute thalamic hyperconnectivity post-mTBI was amplified to an even greater extent by having a history of multiple TBIs. This suggests the neurological effects of repeat injury are cumulative.

Cumulative neurological effects have been consistently reported in animal models of repeat TBI. These have aimed to better understand increased cerebral vulnerability post-injury that can lead to greater adverse effects and neuropathological cascades than a single injury. Such cumulative effects include neurometabolic and lipidomic dysregulation associated with decreased sensorimotor performance,^19^ persistent neuroinflammation associated with cognitive impairment and depression^14^ and thalamic calcium influx.^11^ In one of the few studies to evaluate this in humans, Vagnozzi *et al.*^20^ quantified cerebral *N*-acetylaspartate (NAA) using proton magnetic resonance spectroscopy as an established biochemical marker of brain metabolic imbalance. A short-interval second concussive event produced further decreases of NAA beyond that of single-concussed athletes, extending the recovery of NAA back to baseline levels by 15 days.^20^ While this double concussion group only included three individuals, it demonstrated the cumulative and extended neuropathological cascades of repetitive injury, which can prolong recovery times and exacerbate adverse effects of concussion.

To the best of our knowledge, this exacerbated neuropathology following repetitive injury compared to single injury has not been previously shown in humans using functional neuroimaging, as found here. We thus propose increased thalamocortical functional connectivity is a potential biomarker for a vulnerable neural environment in cases of repetitive injury, as previously found in single mTBI in humans^12^ and a translational rat model.^13^ These studies further established an association between acute thalamocortical hyperconnectivity and persistent PCSs. We did not find poorer functional or post-concussive outcomes in this small cohort of repeat injury to further assess the clinical implication of acute thalamic hyperconnectivity; however, such behavioural effects are well documented in larger samples.^1-5^

Interestingly, identical nuclei were found to present exacerbated cortical hyperconnectivity after repeat mTBI, as those found in single mTBI,^12^ namely, the vAnterior and vlDorsal groups. These nuclei are highly GABAergic,^21^ and thus, amplified hyperconnectivity may be associated with thalamic GABA-related inhibitory imbalance. In support, white matter damage has been documented in the thalamic reticular nucleus, associated with increased thalamocortical coherence in rats following mTBI.^13^ The thalamic reticular nucleus forms an inhibitory GABAergic mesh around the thalami to locally inhibit thalamic activity alongside inhibitory interneurons.^22^ Thus, thalamocortical function and its relationship to excitatory–inhibitory imbalance remains an important field in translational neuroscience for mTBI, and how this can be harnessed in future therapies. Indeed, emerging therapies enhancing GAT-3 in thalamic astrocytes have shown promise in improving outcome in rodents.^23^

Importantly, this study included participants from the public rather than specific elite sporting or military populations. While research in these groups is undeniably significant, the data they provide may not be directly extrapolatable to the wider population of recreational sports players. Here, participants presented merely two or more previous TBIs, of which the majority reported two prior injuries, with no evidence of structural damage resulting from this injury or previous injuries, no neuropsychiatric history and were largely young adults. Such characteristics are commonly associated with a positive recovery post-injury.^24^

Our results have a several limitations to be discussed. Primarily, information was not available regarding the severity of previous injuries. These are inferred to be consistent with mTBI given the lack of CT damage to suggest a more severe injury; however, we cannot be certain whether self-reported injuries reached clinical diagnosis of mTBI. However, this arguably strengthens our results that even these mild repetitive injuries can induce vulnerable neuronal environments. Our interpretation of these results suggests that amplified thalamocortical functional connectivity occurs as a consequence of TBI; however, it could alternatively reflect a risk factor for TBI. Previous study^12^ supports the former, as acute thalamocortical connectivity was significantly increased compared to healthy controls only in those mTBI patients with chronic PCSs. Future study should nevertheless further explore this interpretation and risk factors preceding TBI. Additionally, no inter-injury interval was recorded in these patients or their age at first-time injury or time since most recent mTBI prior to the injury at time of recruitment, limiting our ability to probe windows of vulnerability to cumulative neuropathological effects. Finally, many of the repeat injury group reported sports-related injury. We did not further investigate sports- versus non–sports-related injuries due to limited sample sizes; however, biomechanics of injury type, such as rapid acceleration–deceleration or rotational forces, could impact specific thalamic damage to be explored in future study.

Thus, we have shown how even a few mild repetitive injuries in a non-specialist and otherwise healthy population can induce an exacerbated vulnerable neuronal environment, centred on the thalamus. These results further establish thalamocortical functional connectivity as a scalable marker of acute injury and long-term neural dysfunction following mTBI.

## Supplementary Material

fcae223_Supplementary_Data

## Data Availability

The data that support the findings of this study can be accessed by application at CENTER-TBI (https://www.center-tbi.eu/data). Codes generated and used within this work have been uploaded at https://github.com/rebeccaemma24/Repeat_mTBI_thalamic_hyperconnectivity.

## Appendix I CENTER-TBI MRI sub-study participants and investigators

Krisztina Amrein, Nada Andelic, Lasse Andreassen, Audny Anke, Philippe Azouvi, BoMichael Bellander, Habib Benali, Andras Buki, Alessio Caccioppola, Emiliana Calappi, Marco Carbonara, Giuseppe Citerio, Hans Clusmann, Mark Coburn, Jonathan Coles, Marta Correia, Endre Czeiter, Véronique De Keyser, Vincent Degos, Bart Depreitere, Live Eikenes, Erzsébet Ezer, Kelly Foks, Shirin Frisvold, Damien Galanaud, Alexandre Ghuysen, Ben Glocker, Asta Haberg, Iain Haitsma, Eirik Helseth, Peter J. Hutchinson, Evgenios Kornaropoulos, Noémi Kovács, Ana Kowark, Steven Laureys, Didier Ledoux, Hester Lingsma, Andrew I. R. Maas, Geoffrey Manley, David K. Menon, Tomas Menovsky, Benoit Misset, Visakh Muraleedharan, Ingeborg Nakken, Virginia Newcombe, Wibeke Nordhøy, József Nyirádi, Fabrizio Ortolano, Paul M. Parizel, Vincent Perlbarg, Paolo Persona, Wilco Peul, Jussi P. Posti, Louis Puybasset, Sophie Richter, Cecilie Roe, Olav Roise, Rolf Rossaint, Sandra Ross, Daniel Rueckert, Ranjit D. Singh, Toril Skandsen, Abayomi Sorinola, Emmanuel Stamatakis, Ewout W. Steyerberg, Nino Stocchetti, Riikka Takala, Viktória Tamás, Olli Tenovuo, Aurore Thibaut, Zoltán Vámos, Gregory Van der Steen, Inge A. van Erp, Wim Van Hecke, Thijs Vande Vyvere, Jan Verheyden, Anne Vik, Victor Volovici, Lars T. Westlye, Daniel Whitehouse, Guy Williams, Stefan Winzeck, Peter Ylén and Tommaso Zoerle.
