# Supplementary material for: Repeat traumatic brain injury exacerbates acute thalamic hyperconnectivity in humans
Source: Brain Commun. 2024 Jun 28;6(4):fcae223. doi: 10.1093/braincomms/fcae223 (PMC11235327; doi:10.1093/braincomms/fcae223)

Supplementary Materials

Supplementary Methods 1. Preprocessing of neuroimaging data.

Preprocessing was largely performed using fMRIprep[1] (v1.5.4). This combines best-judged aspects of different software for a standardised and freely accessible preprocessing pipeline.

Firstly, T1-weighted structural MRI data were corrected for non-uniformity in image intensity using N4BiasFieldCorrection, distributed with ANTs 2.2.0. These images were then skull-stripped, also using ANTs, and segmented into three tissue classes using FSL's FAST (FSL 5.0.9). These were white-matter, grey-matter, and cerebrospinal fluid. Structural data were then spatially normalised to the ICBM 152 Nonlinear Asymmetrical template version 2009c (MNI152NLin2009cAsym) standard-space template using nonlinear registration with antsRegistration (ANTs 2.2.0), using brain-extracted versions of the T1-weighted reference image for each individual and the T1 template. This was applied to bring all images into an identical space for group-level analysis.

Next, a reference volume of each rsfMRI image was generated and skull-stripped using a custom methodology of *fMRIprep*. This reference image was subsequently coregistered to the T1-weighted reference image (in subject-space) using FLIRT (FSL 5.0.9) with boundary-based registration cost-function. This used 9 degrees of freedom to account for distortions remaining in the rsfMRI reference image. Head motion was then estimated by applying calculated transforms to correct for 6 directions of rotation and translation calculated using MCFLIRT (FSL 5.0.9). This was applied in subject space by resampling. Corrected images were then normalised into standard space, using the previously calculated transformations applied to T1-weighted images.

Additionally, functional data were denoised by signal regression and spatially smoothed with a 6mm gaussian kernel. Denoising included covariates of average white matter and cerebrospinal fluid timeseries', rigid-body head motion (12 DOF), and temporal high-pass filter. These are estimated within fMRIprep, whereby high-pass filtering uses a General Linear Model with the Discrete Cosine Transformation approach to produce a basis of discrete cosines for frequencies slower than 0.008 Hz. Any volumes identified as motion outliers (exceeded 0.5 mm framewise displacement or 1.5 standardised DVARS) were removed from the data, and any data exceeding n=164 volumes were also removed from the end of acquisition for group-

level analysis. No subjects were removed entirely following censoring, as all presented over 4 minutes of uncontaminated data[2].

1. Esteban O, Markiewicz CJ, Blair RW, et al. fMRIPrep: a robust preprocessing pipeline for functional MRI. *Nat Methods*. 2019;16(1):111-116. doi:10.1038/s41592-018-0235-4
2. Parkes L, Fulcher B, Yücel M, Fornito A. An evaluation of the efficacy, reliability, and sensitivity of motion correction strategies for resting-state functional MRI. *Neuroimage*. 2018;171:415-436. doi:10.1016/J.NEUROIMAGE.2017.12.073

Supplementary Figure 1. Consort diagram for patient inclusion. All data were obtained from CENTER-TBI (vCORE 3.0).
GCS = Glasgow Coma Scale, mTBI = mild traumatic brain injury, rsfMRI = resting-state functional magnetic resonance imaging, ICU = intensive care unit, CENTER-TBI = Collaborative European NeuroTrauma Effectiveness Research in Traumatic Brain Injury.

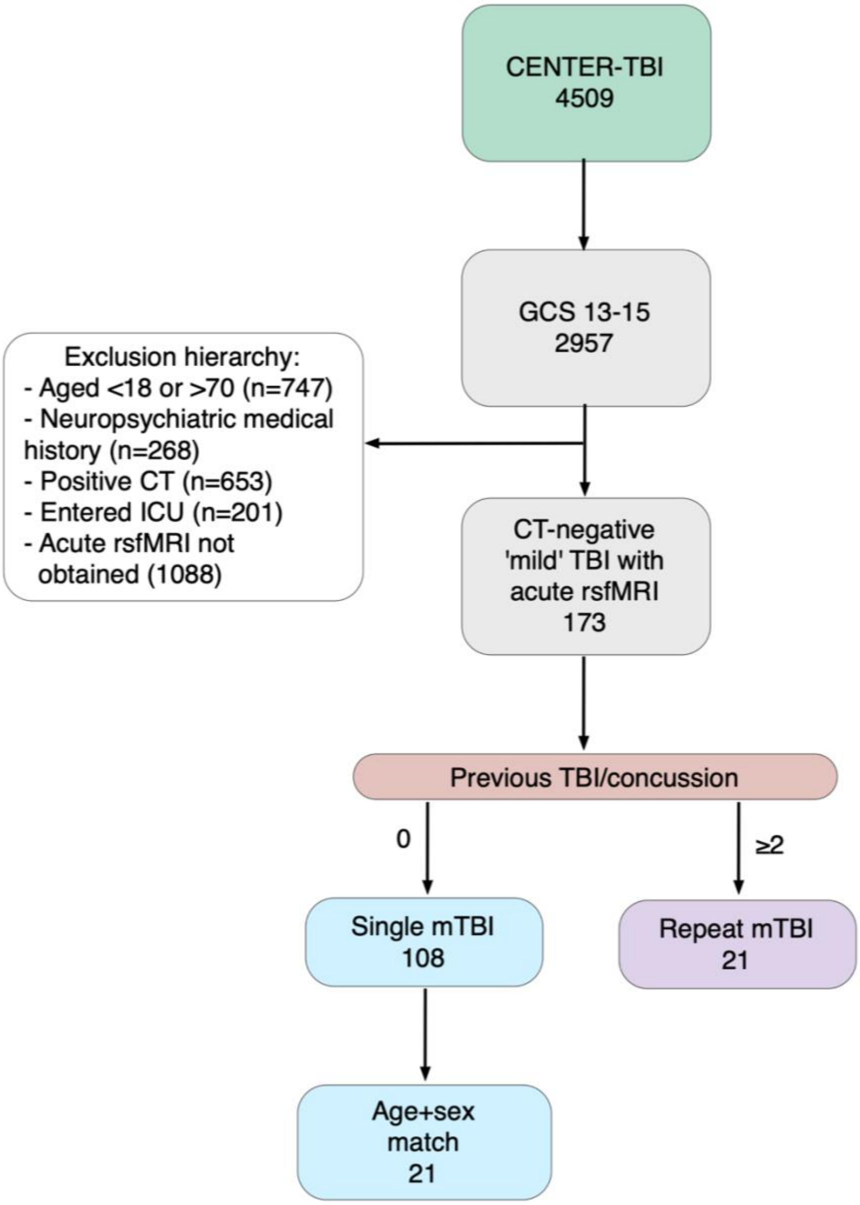

Supplementary Table 1. Thalamocortical functional connectivity between groups. Values were compared for increasing thalamocortical connectivity across the three groups (control, single mTBI, repeat mTBI) using a one-tailed Jonckheere-Terpstra test for non-parametric rank-based trends, with N=1000 permutations. All tests were adjusted for effects of age and sex, and FDR-corrected at p<0.05. Significant variables are denoted in bold.

**Thalamic ROI**	**Jonckheere-Terpstra**
**Left Thalamus**	**T_{JT} = 2244, *p* = .022**
Right Thalamus	T_{JT} = 2111, *p* = .12
Left Hemisphere Nuclei	
Pulvinar	T_{JT} = 1896, *p* = .36
Anterior	T_{JT} = 2120, *p* = .12
mDorsal	T_{JT} = 2030, *p* = .18
**vlDorsal**	**T_{JT} = 2375, *p* = .008**
Central	T_{JT} = 2003, *p* = .23
**vAnterior**	**T_{JT} = 2399, *p* = .008**
vlVentral	T_{JT} = 2135, *p* = .10
Right Hemisphere Nuclei	
Pulvinar	T_{JT} = 1703, *p* = .72
Anterior	T_{JT} = 2068, *p* = .13
mDorsal	T_{JT} = 2023, *p* = .18
**vlDorsal**	**T_{JT} = 2291, *p* = .020**
Central	T_{JT} = 1910, *p* = .35
**vAnterior**	**T_{JT} = 2358, *p* = .011**
vlVentral	T_{JT} = 1856, *p* = .42

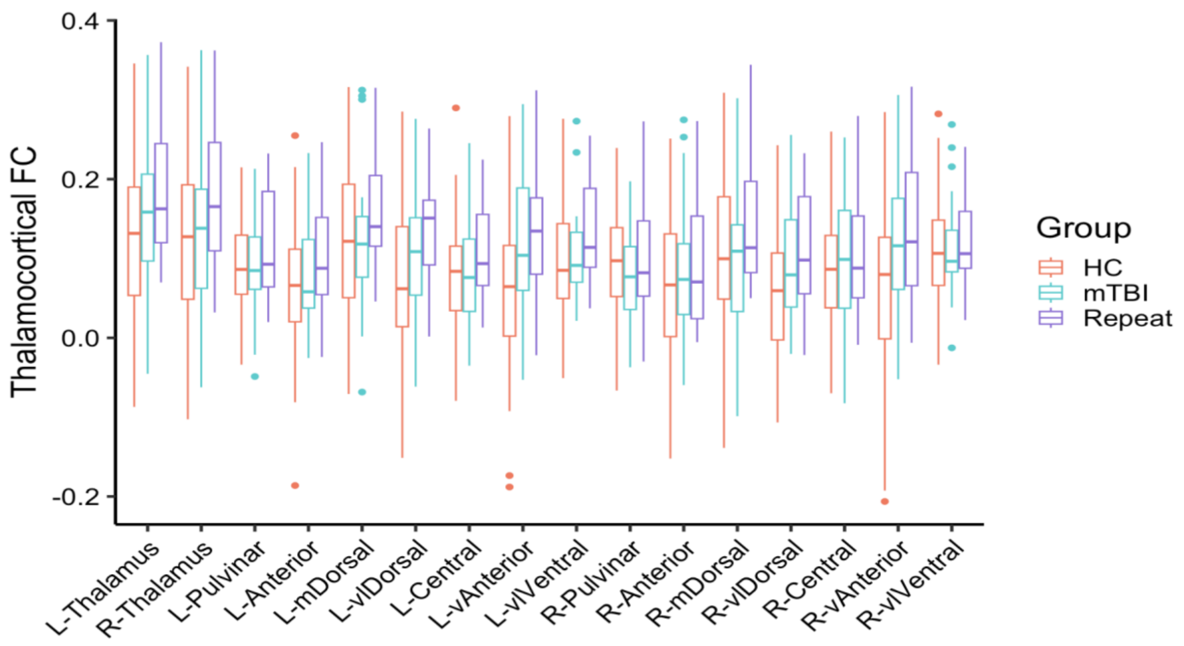

Supplement: fcae223_Supplementary_Data [file fcae223_supplementary_data.pdf]
